# Preoperative neutrophil-to-lymphocyte ratio is a predictor of survival of epithelial ovarian cancer: a systematic review and meta-analysis of observational studies

**DOI:** 10.18632/oncotarget.16793

**Published:** 2017-04-03

**Authors:** Zhuo Yang, Jia-Hui Gu, Cui-Shan Guo, Xin-Hui Li, Wen-Chao Yang

**Affiliations:** ^1^ Department of Obstetrics and Gynecology, Shengjing Hospital of China Medical University, Shenyang, China

**Keywords:** neutrophil, lymphocyte, ovarian cancer, survival, meta-analysis

## Abstract

Inflammation plays an important role in the development and progression of epithelial ovarian cancer (EOC). However, no meta-analysis has comprehensively and quantitatively investigated the prognostic value of the neutrophil-to-lymphocyte ratio (NLR) in EOC patients. Therefore, we performed a meta-analysis to quantify the prognostic impact of this biomarker. We searched the PubMed and Web of Science databases from their inception through December 31, 2016, and examined observational studies evaluating the association of preoperative NLR with progression-free survival (PFS) and overall survival (OS) of EOC patients. A random-effects model was used to summarize hazard ratios (HRs) with 95% confidence intervals (CIs). Twelve retrospective cohort studies including 3,154 EOC patients were identified. Elevated NLR in EOC patients was associated with worse PFS (summarized HR=1.80; 95% CI = 1.22–2.65; *I*^2^ = 79.1%) and OS (summarized HR = 1.72; 95% CI = 1.18–2.51; *I*^2^ = 73.5%) compared with low NLR. No evidence of publication bias was detected by funnel plot analysis and formal statistical tests. Although the results were robust in all subgroup analyses, not all results were statistically significant. We determined that adjustments for CA-125 level and performance status might be sources of heterogeneity. These combined results indicate that preoperative NLR is an important predictor of prognosis in EOC patients. Since the high heterogeneity and retrospective study design of included studies, these results require further validation with prospective cohort and trials enrolling larger patient populations and conducting longer follow-up examinations.

## INTRODUCTION

Epithelial ovarian cancer (EOC) is the second-most common gynecological cancer in the world; globally, there were approximately 0.23 million new cases and 0.15 million deaths in 2012 [1]. Primary cytoreductive surgery and platinum-based adjuvant chemotherapy remains the gold standard in treatment for EOC patients [2]. However, the majority of these patients are diagnosed at a later stage, half of the patients experience recurrence within 16 months, and the 5-year overall survival rate is below 50% [3–6]. The International Federation of Gynecology and Obstetrics (FIGO) established several predictors of survival for EOC patients, including age at diagnosis, stage, histological grade, residual tumor, ascites, performance status (PS), and cancer antigen 125 (CA-125) levels [7, 8]. However, effective biomarkers for individualized prediction of treatment outcomes and prognosis are still urgently required [4].

Inflammation is involved in all stages of cancer formation, including initiation, promotion, development, and progression of EOC [9]. Therefore, systemic inflammatory response (SIR) markers such as hypoalbuminemia, hyperfibrinogenemia, C-reactive protein, absolute white blood cell count, neutrophil-to-lymphocyte ratio (NLR), and platelet-to-lymphocyte ratio have been investigated as prognostic factors in cancer patients [10–13]. Among these biomarkers, NLR is a combination of peripheric neutrophils and lymphocytes, which represents the host systemic inflammatory response and the immunity status of the patient [14]. Previous studies hypothesized that increased NLR is associated with some unfavorable clinico-pathological features and poor survival of EOC patients [15–17]. However, the relationship between NLR and survival of EOC patients is not consistent in epidemiological studies. Several studies reported that preoperative peripheral blood NLR is an independent predictor of poor prognosis in ovarian cancer [14, 18–24], whereas other studies failed to find any evidence of this association [4, 15–17].

To the best of our knowledge, no systematic review and meta-analysis has investigated the prognostic value of NLR as a predictive biomarker for patients with EOC. The purpose of this study was to summarize the evidence from observational studies for an association of preoperative NLR with progression-free survival (PFS) and overall survival (OS) in EOC patients from observational studies.

## RESULTS

### Search results, study characteristics, and quality assessment

**Figure 1:**
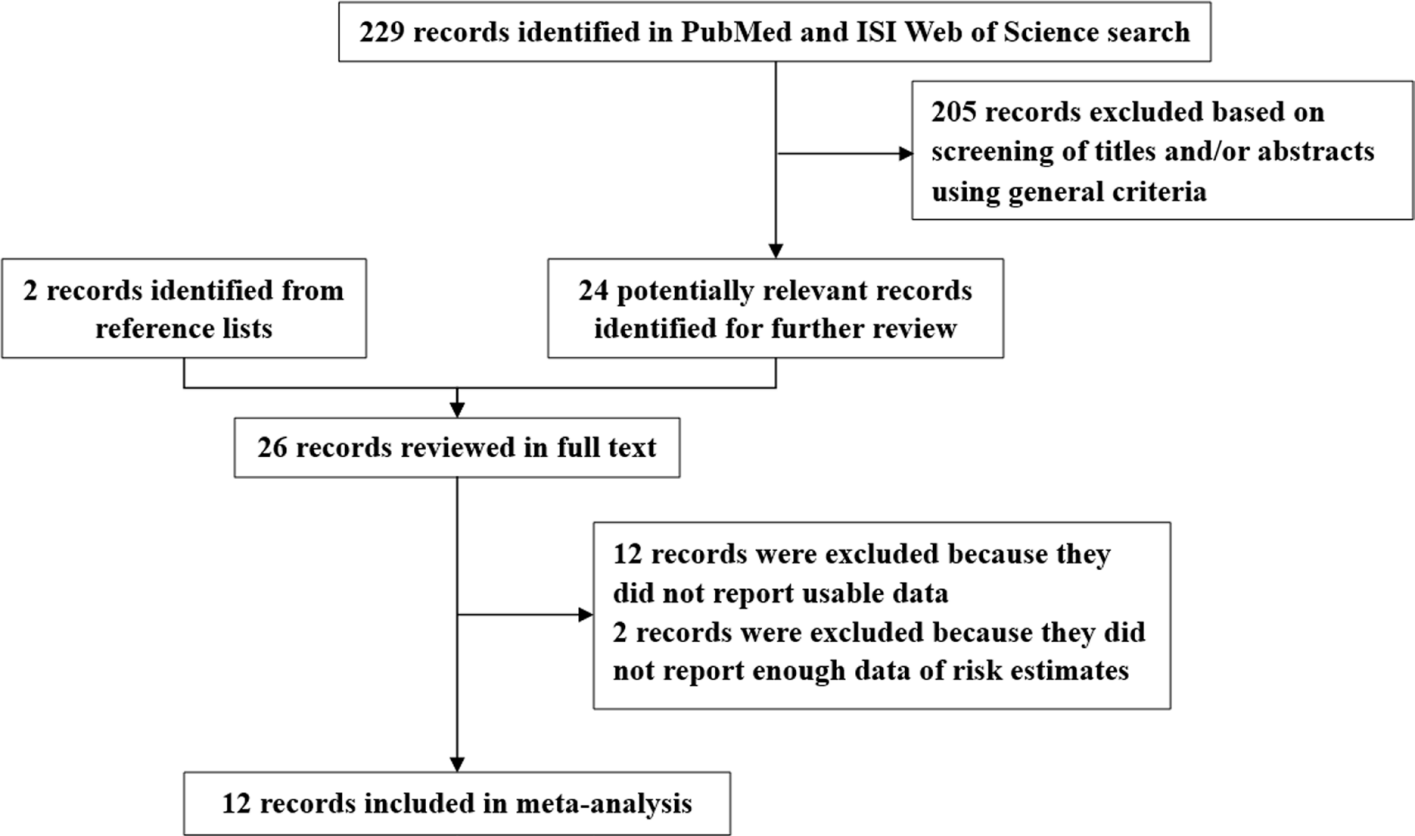
Selection of studies for inclusion in this meta-analysis

The detailed processes of literature screening, study selection, and study exclusion are summarized in Figure 1. Our initial search retrieved 229 unique reports. After removing duplicates and screening the titles and abstracts, 35 articles were considered as potentially eligible for inclusion, and were subjected to full-text review. After exclusion, 12 observational studies were included in the meta-analysis [4, 14–24].

Table 1 presents the main characteristics of the 12 included studies. These studies were published from 2009 to 2016, and included a total of 3, 154 ovarian cancer patients with a range of 30–875 cases in individual studies. These 12 reports were designed as retrospective cohort studies. The majority of the included studies were conducted in Asia (*n* = 9) [4, 14, 15, 17, 19–22, 24], two were conducted in Europe [16, 18], and one was conducted in the United States [23]. Ten studies [4, 14–17, 19–22, 24] treated NLR as a categorical variable, whereas two studies [18, 23] treated NLR as a continuous variable. The cut-off values among the 10 studies were defined as follows: 6 studies utilized receiver-operating curve (ROC) [14, 15, 18–20, 22, 24], one study utilized median levels [4] and one study utilized interquartile levels [21], one study utilized the cut-off value defined by a previous study [17], and one study failed to mention this issue [16]. More than half of the included studies adjusted for age at diagnosis/surgery (*n* = 8), FIGO stage (*n* = 7), and CA-125 level (*n* = 6). Fewer studies adjusted for SIR markers (*n* = 5), tumor grade (*n* = 4), and PS (*n* = 3).

**Table 1:** Characteristics of twelve retrospective cohort studies included in the meta-analysis

First author, [ref], year, country	No. of cases	Exposure characteristics	Outcome	Hazard ratio (95% CI)	Adjustment for potential confounders					
					Age	FIGO	Grade	PS	CA-125	SIR markers
Badora-Rybicka et al. [18], 2016, Poland	315	Continuous	PFS OS	1.22 (1.08–1.38) 1.09 (0.98–1.22)	√	√		√	√	
Eo et al. [19], 2016, Korea	234	Category (≤ 4.28 vs. > 4.28) (≤ 5.03 vs. > 5.03)	PFS OS	2.34 (1.45–3.77) 2.01 (1.18–3.43)						
Feng et al. [4], 2016, China	875	Category (≤ 3.24 vs. > 3.24)	PFS OS	1.25 (1.05–1.48) 1.19 (0.94–1.50)	√	√				
Nakamura et al. [20], 2016, Japan	30	Category (≤ 3.91 vs. > 3.91)	OS	14.1 (1.21–165)	√			√	√	
Wang et al. [14], 2016, China	143	Category (≤ 3.43 vs. > 3.43)	PFS OS	3.37 (1.39–8.15) 2.20 (1.03–4.70)	√	√			√	√
Wang et al. [21], 2015, China	126	Category (≤ 1.86 vs. > 3.77)	PFS OS	6.87 (2.64–17.9) 8.57 (2.81–26.1)	√	√	√	√	√	√
Zhang et al. [22], 2015, China	190	Category (≤ 3.4 vs. > 3.4)	PFS OS	2.01 (1.48–2.74) 2.17 (1.55–3.05)						
Williams et al. [23], 2014, USA	519	Continuous	OS	1.37 (1.06–1.76)	√	√	√		√	
Raungkaewmanee et al. [15], 2012, Thailand	166	Category (≤ 2.6 vs. > 2.6)	PFS OS	1.12 (0.61–2.07) 1.17 (0.63–2.19)						
Asher et al. [16], 2011, United Kingdom	235	Category (≤ 4 vs. > 4)	OS	0.87 (0.52–1.44)	√		√			√
Thavaramara et al. [17], 2011, Thailand	129	Category (≤ 2.6 vs. > 2.6)	PFS OS	0.7 (0.3–1.4) 0.7 (0.3–1.6)		√				√
Cho et al. [24], 2009, Korea	192	Category (≤ 2.6 vs. > 2.6)	OS	8.42 (1.09–64.8)	√	√	√		√	√

Abbreviations: BMI, body mass index; CA-125, carbohydrate antigen-125; CI, confidence interval; FIGO, International Federation of Gynecology and Obstetrics; N/A, not available; PFS, progression-free survival; PS, performance status; OS, overall survival; SIR, systemic inflammatory response.

The quality assessment characteristics of the included studies are shown in Table 2. The major difference among these included studies was the control for an important factor or an additional factor category; 7 of the included studies were assigned two full scores [4, 14, 16, 18, 21, 23, 24]. The study by Nakamura et al. [20] only reported mortality within 100 days; therefore, this study was not assigned a score when testing for whether the follow-up duration was long enough for outcomes to occur.

**Table 2:** Methodological quality of twelve retrospective cohort studies included in the meta-analysis

sFirst author (reference), year	Representativeness of the exposed cohort	Selection of the unexposed cohort	Ascertainment of exposure	Outcome of interest not present at start of study	Control for important factor or additional factor^†^	Assessment of outcome	Follow-up long enough for outcomes to occur^‡^	Adequacy of cohort follow-up^§^
Badora-Rybicka [18], 2016	*	*	*	*	**	*	*	*
Eo [19], 2016	*	*	*	*	-	*	*	*
Feng [4], 2016	*	*	*	*	**	*	*	*
Nakamura [20], 2016	*	*	*	*	*	*	-	*
Wang [14], 2016	*	*	*	*	**	*	*	*
Wang [21], 2015	*	*	*	*	**	*	*	*
Zhang [22], 2015	*	*	*	*	-	*	*	*
Williams [23], 2014	*	*	*	*	**	*	*	*
Raungkaewmanee [15], 2012	*	*	*	*	-	*	*	*
Asher [16], 2011	*	*	*	*	**	*	*	*
Thavaramara [17], 2011	*	*	*	*	*	*	*	*
Cho [24], 2009	*	-	*	*	**	*	*	*

A study could be awarded a maximum of one star for each item except for the item “Control for important factor or additional factor.” The definition/explanation of each column of the Newcastle-Ottawa Scale is available from (http://www.ohri.ca/programs/clinical_epidemiology/oxford.asp).

^†^ A maximum of two stars could be awarded for this item. Studies that controlled for age at diagnosis, International Federation of Gynecology and Obstetrics (FIGO) stage received one star, whereas studies that controlled for other important confounders such as residual disease received an additional star.

^‡^ A cohort study with a median follow-up time > 24 months was assigned one star.

^§^ A cohort study with a follow-up rate > 75% was assigned one star.

### Progression-free survival

**Figure 2:**
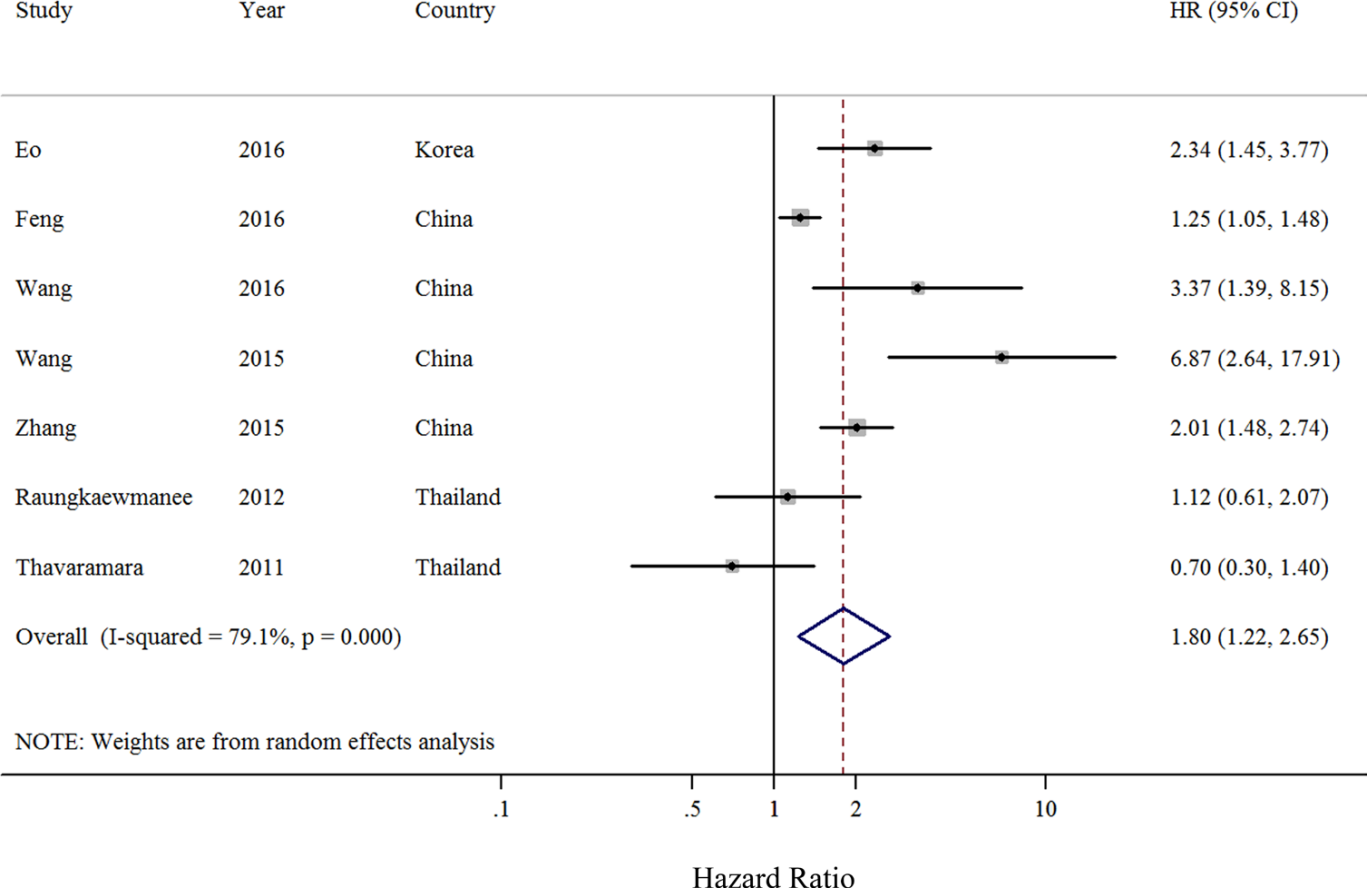
Forest plot (random-effects model) of neutrophil-to-lymphocyte ratio and progression-free survival of patients with ovarian cancer The squares indicate study-specific hazard ratios (size of the square reflects the study-specific statistical weight); the horizontal lines indicate 95% CIs; and the diamond indicates the summary hazard ratio estimate with its 95% CI.

**Figure 3:**
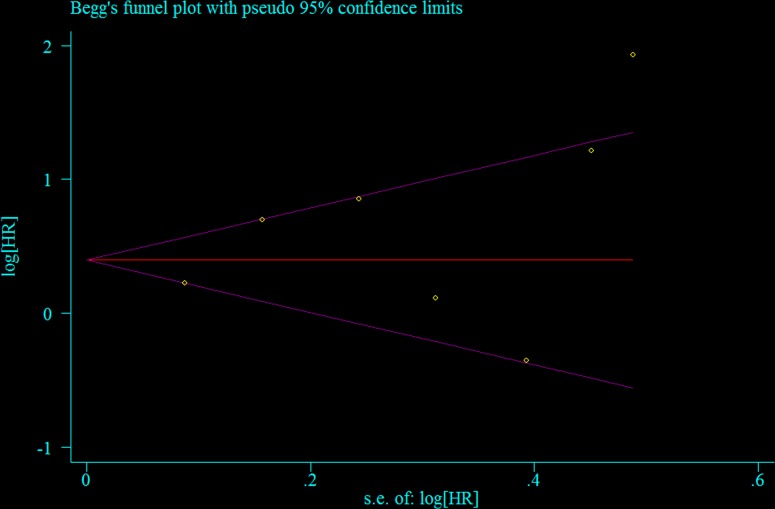
Test for publication bias for progression-free survival through Begg's funnel plot HR, hazard ratio; SE, standard error.

Seven retrospective cohort studies including a total of 1,863 patients evaluated the association between NLR and PFS of ovarian cancer [4, 14, 15, 17, 19, 21, 22]. Compared with the low NLR, elevated NLR indicated worse PFS in ovarian cancer patients (summarized HR = 1.80; 95% CI = 1.22–2.65) (Figure 2), with significant heterogeneity (*I*^2^ = 79.1%) (Supplementary Figures 1 and 2). No evidence of publication bias was indicated by funnel plot analysis (Figure 3) and formal statistical tests (Egger test, *P* = 0.26; Begg test, *P* = 0.55). The results of subgroup analyses are presented in Table 3. Although the direction of all subgroup analyses consistently indicated that poor PFS was associated with ovarian cancer, not all of them showed statistically significance. Significant results were obtained for differences between adjustments for CA-125 levels in meta-regression analyses. Our sensitivity analysis was performed by excluding one study at a time. The sensitivity analysis showed that the summarized HR for PFS ranged from 1.57 (95% CI = 1.11–2.21; *I*^2^ = 3.2%; exclusion of Wang et al. [21]) to 2.03 (95% CI = 1.36–3.03; *I*^2^ = 79.9%; exclusion of Thavaramara et al. [17]).

**Table 3:** Risk estimates summary of the association of neutrophil-to-lymphocyte ratio with progression-free and overall survival of ovarian cancer patients

	Progression free survival						Overall survival					
	No. of	HR	95%	*I*^2^ (%)	Ph^†^	Ph^‡^	No. of	HR	95%	*I^2^* (%)	Ph^†^	Ph^‡^
	Study		CI				Study		CI			
**Overall**	7	1.80	1.22–2.65	79.1	< 0.01		10	1.72	1.18–2.51	73.5	< 0.01	
**Subgroup analyses**												
**Number of cases**						0.53						0.41
≥ 150	4	1.61	1.14–2.27	74.4	< 0.01		6	1.48	1.04–2.12	70.0	< 0.01	
< 150	3	2.47	0.63–9.672	86.5	< 0.01		4	2.94	0.87–9.96	80.1	< 0.01	
**Geographic location**						N/A						0.35
Asia	7	1.80	1.22–2.65	79.1	< 0.01		9	1.91	1.27–2.86	72.8	< 0.01	
Europe	0	N/A	N/A	N/A	N/A		1	0.87	0.52–1.44	N/A	N/A	
**Cut-off method**						0.75						0.37
ROC	4	1.98	1.41–2.78	42.4	0.16		6	2.03	1.44–2.88	32.9	0.19	
Non-ROC	3	1.70	0.64–4.46	86.0	< 0.01		4	1.36	0.71–2.59	80.0	< 0.01	
**Adjustment for potential confounders and risk factors**												
**Age at diagnosis/surgery**						0.31						0.39
Yes	3	2.85	0.95–8.55	87.5	< 0.01		6	2.28	1.17–4.44	77.9	< 0.01	
No	4	1.51	0.96–2.39	69.0	0.02		4	1.52	0.97–2.39	62.6	0.05	
**FIGO stage**						0.87						0.73
Yes	4	1.97	0.88–4.43	46.4	0.16		5	2.09	0.98–4.48	78.6	< 0.01	
No	3	1.84	1.29–2.63	84.0	< 0.01		5	1.60	0.99–2.59	69.7	0.01	
**Tumor grade**						0.09						0.44
Yes	1	6.87	2.64–17.91	N/A	N/A		3	3.52	0.57–21.94	87.8	< 0.01	
No	6	1.57	1.11–2.21	73.2	< 0.01		7	1.57	1.10–2.22	65.6	< 0.01	
**Performance status**						0.09						0.02
Yes	1	6.87	2.64–17.91	N/A	N/A		2	9.33	3.38–25.77	0	0.72	
No	6	1.57	1.11–2.21	73.2	< 0.01		8	1.44	1.04–1.99	66.2	< 0.01	
**CA-125 level**						0.04						0.02
Yes	2	4.70	2.34–9.42	12.8	0.28		4	5.18	2.01–13.38	47.3	0.13	
No	4	1.45	1.03–2.05	73.4	< 0.01		6	1.32	0.94–1.84	69.2	< 0.01	
**SIR markers**						0.53						0.90
Yes	3	2.47	0.63–9.672	86.5	< 0.01		5	2.05	0.83–5.04	80.4	< 0.01	
No	4	1.61	1.14–2.27	74.4	< 0.01		5	1.66	1.12–2.46	69.9	0.01	

Abbreviations: CA-125, carbohydrate antigen-125; CI, confidence interval; FIGO, International Federation of Gynecology and Obstetrics; HR, hazard ratio; N/A, not available; ROC, receiver-operating curve; SIR, systemic inflammatory response.

^†^*P*-value for heterogeneity within each subgroup.

^‡^*P*-value for heterogeneity between subgroups with meta-regression analysis.

### Overall survival

**Figure 4:**
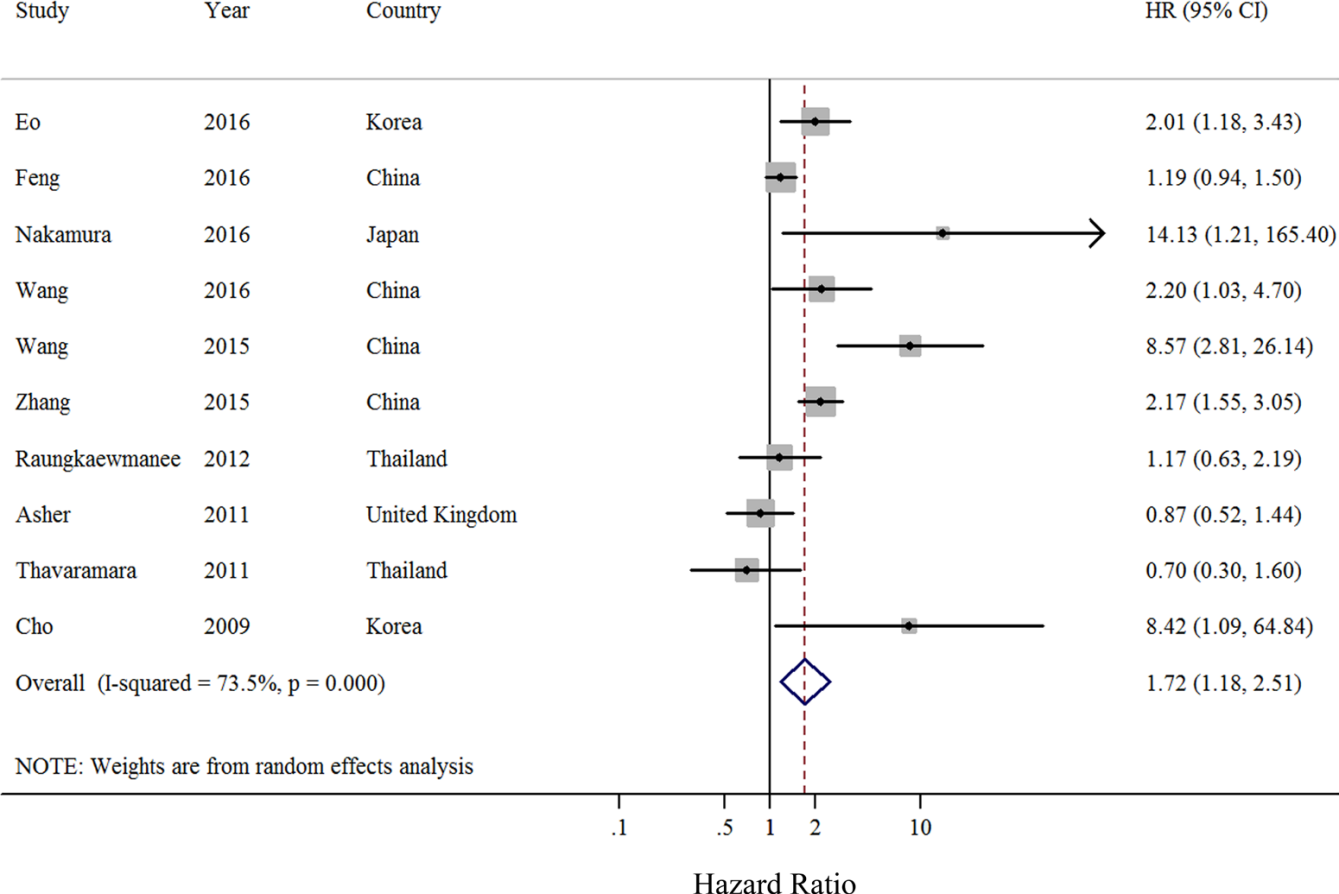
Forest plot (random-effects model) of neutrophil-to-lymphocyte ratio and overall survival of patients with ovarian cancer The squares indicate study-specific hazard ratio (size of the square reflects the study specific statistical weight); the horizontal lines indicate 95% CIs; and the diamond indicates the summary hazard ratio estimate with its 95% CI.

**Figure 5:**
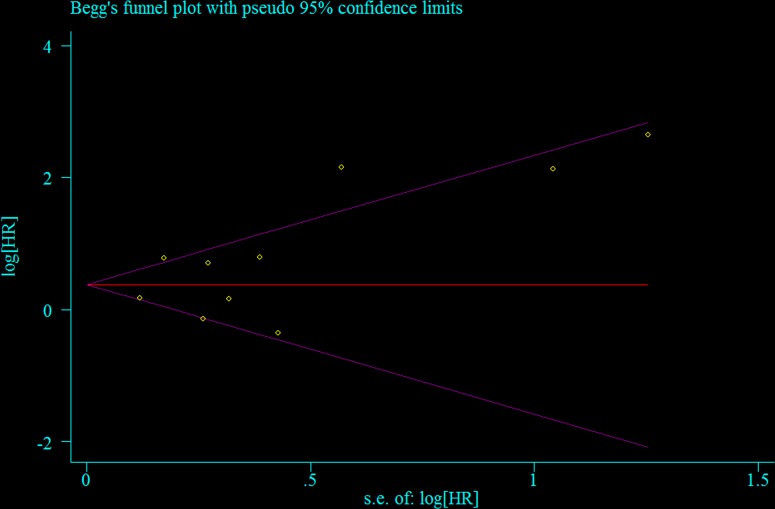
Test for publication bias for overall survival through Begg's funnel plot Abbreviations: HR, hazard ratio; SE, standard error.

Eleven retrospective cohort studies including a total of 2, 320 patients evaluated the association of NLR with OS of patients with ovarian cancer [4, 14–17, 19–22, 24]. Compared with low NLR, elevated NLR indicated worse OS in ovarian cancer patients (summarized HR = 1.72; 95% CI = 1.18–2.51) (Figure 4), with significant heterogeneity (*I*^2^ = 73.5%) (Supplementary Figure 1). No evidence of publication bias was indicated by visual inspection of a funnel plot analysis (Figure 5) and formal statistical tests (Egger test, *P* = 0.16; Begg test, *P* = 0.15). The results of subgroup analyses are presented in Table 3. Although the direction of all subgroup analyses consistently indicated that poor OS was associated with ovarian cancer, not all of them showed statistically significance. Significant results were obtained for differences between adjustments for performance status and CA-125 level in the meta-regression analysis. Our sensitivity analysis was performed by excluding one study at a time, and the results indicated that the summarized HR for OS ranged from 1.50 (95% CI = 1.07–2.11; *I*^2^ = 66.8%; exclusion of Wang et al. [21]) to 1.91 (95% CI = 1.27–2.86; *I*^2^ = 72.8%; exclusion of Asher et al. [16]).

## DISCUSSION

To the best of our knowledge, this is the first systematic review and meta-analysis focusing on the prognostic value of NLR in patients with EOC. The results of this study indicate that, compared with low NLR, elevated NLR was associated with worse PFS and OS in patients with EOC.

The exact mechanisms underlying the association of elevated NLR with the prediction of poor survival in EOC patients have not been determined. Accumulating evidence suggests that inflammation is involved in the initiation, promotion, and progression of cancer [9]. Some studies reported that neutrophils and lymphocytes have prominent roles in inflammatory and immunological responses to tumors [25, 26]; however, they have different functions in the inflammatory response. Neutrophils are the primary source of circulating vascular endothelial growth factor (VEGF), which contributes to tumor-related angiogenesis [14, 27, 28]. Neutrophils also suppress the cytolytic activity of CD8+ T-lymphocyte cells, which effectively delay tumor progression similarly as natural killer cells, and regulate T-cell activation [29, 30]. This creates an immunosuppressive milieu, which is beneficial to tumorigenesis [31, 32]. Conversely, lymphocytes induce cytotoxic cell death and suppress tumor cell proliferation and migration, which are important for defense against cancer proliferation and development [19]. Previous studies have reached a consensus that tumor-infiltrating lymphocytes establish a defense barrier against cancer metastasis [9, 33]. NLR represents a balance between host immunity and tumor angiogenesis, and elevation of NLR would trigger an angiogenic response favorable to tumor cells [14].

Although 10 of the 12 included studies treated NLR as a categorical variable, the cut-off value for NLR varied among these studies due to methodological differences. For example, seven of the included studies [14, 15, 18–20, 22, 24] optimized NLR cut-off values for outcomes using ROC values ranging from 2.6 to 5.03. By contrast, one study used a median level (3.24) [4] and one study used an interquartile level (1.86–3.77, from the lowest to highest category) [21]. We could not distinguish which method was most accurate, and did not find that the cut-off method was a source of heterogeneity in the meta-regression analysis (Table 3). Nevertheless, the heterogeneity between these two groups was obviously different (42.4% *vs*. 86%; 32.9% *vs*. 80%). Future studies are needed to clarify which cut-off method provides the most accurate values for estimating prognostic risk for patients with EOC.

The strengths of our meta-analysis include a large sample size (3154 EOC patients) and no significant evidence of publication bias. All of the included studies were published within 8 years (2009 to 2016), and all had performed consistent experimental procedures for measuring NLR. The results of our subgroup and sensitivity analyses were consistent, which suggested that the results were robust. This meta-analysis has several limitations that should be acknowledged. First, the 12 included studies were all designed as retrospective cohort studies, which depended on medical records or documentation and avoided potential recall bias. The results significantly differed according to whether PS and CA-125 levels were adjusted, which suggested that PS and CA-125 levels might be sources of heterogeneity, although we could not rule out the possibility that the limited number of studies for the subgroup analyses might have introduced heterogeneity. Second, 8 meeting abstracts [34–41] failed to provide HR and 95% CI, or sufficient data to calculate these parameters, and were excluded from the final analysis. Although this limited the sample size of the present meta-analysis, the inclusion of meeting abstracts may lower the meta-analysis quality because they have not undergone rigorous peer review [13]. Third, NLR is associated with many factors such as cigarette smoking, use of non-steroidal anti-inflammatory drugs, chronic infection or inflammatory disease, and PS [20, 23, 42, 43]. The retrospective cohort design and the limited number of included studies precluded adjustment for these potential confounders in the primary analyses. Therefore, it is possible that the association between NLR and survival of EOC could result from unmeasured or residual confounding effects due to these factors. Fourth, only two of the included studies [18, 23] treated NLR as a continuous variable in the primary multivariate analyses; therefore, we could not evaluate dose-response associations between NLR and survival of EOC patients. Further studies are warranted to obtain sufficient data or conduct dose-response analyses in the future.

In conclusion, the first systematic review and meta-analysis of observational studies conducted a comprehensive and quantitative investigation into the association between NLR and survival of EOC patients. We found that increased NLR was significantly associated with worse PFS and OS in patients with EOC, compared with low NLR. Since the high heterogeneity which might limit the interpretation of this finding, this result requires further validation with prospective cohort and trials enrolling larger patient populations and conducting longer follow-up examinations. NLR may serve as a readily available and cost-effective prognostic marker in clinical practice for EOC.

## MATERIALS AND METHODS

### Search strategy

Two independent investigators (ZY and J-HG) systematically searched for relevant epidemiological studies published in PubMed (MEDLINE) and Web of Science databases starting from the time of each database's inception to December 31, 2016. The following search keywords and terms were used: (neutrophil-to-lymphocyte OR neutrophil OR lymphocyte OR NLR) AND (ratio) AND (ovary OR ovarian) AND (cancer OR neoplasms OR carcinoma OR tumor). The meta-analysis was planned, conducted, and reported according to the guidelines of the Meta-Analysis of Observational Studies in Epidemiology (MOOSE) group [44].

### Study selection and exclusion

The following inclusion criteria were used: (i) observational study design; (ii) studies evaluated the association of NLR with PFS and OS of ovarian cancer patients; (iii) studies performed hazard ratio (HR) or relative risk (RR) analyses with 95% confidence intervals (CIs), or reported sufficient data to calculate those risk estimates, and (iv) the NLR was defined as the absolute neutrophil count divided by the absolute lymphocyte count. The following exclusion criteria were used: (i) randomized controlled trials, case-control studies, reviews without original data, ecological studies, editorials, and case reports; (ii) studies that reported risk estimates without 95% CI (e.g., studies that could not be included in the statistical summary).

We checked titles and abstracts of retrieved articles for relevancy, and then examined the full-text articles. The relevant data were extracted from the complete articles. We also performed manual scans of the bibliographies of the selected articles. The study selection and exclusion procedures were performed by two independent investigators (ZY and J-HG).

### Data abstraction and quality assessment

The following information was extracted for each included study by a single investigator (ZY): first author, year of publication, country, number of patients, exposure characteristics, outcomes, and study-specific adjusted risk estimates with 95% CIs (including adjusted confounder information if applicable). The predefined primary outcome was progression-free survival, and the secondary outcome was overall survival. Extracted data were entered into a standardized Excel (Microsoft) file. Subsequently, an independent investigator (J-HG) checked the data, and all differences were resolved by a third investigator (C-SG). Two independent investigators (ZY and J-HG) assessed the methodological quality of the included studies according to the Newcastle-Ottawa Scale (NOS) [45–47].

### Statistical analysis

We calculated the summarized HR with 95% CI by summarizing the risk estimates of each study using a random-effect model to investigate the association of NLR with PFS and OS of patients with ovarian cancer. Only two of the included studies provided sufficient data [18, 23]; therefore, we were unable to evaluate the dose-response association of NLR with PFS and OS. Heterogeneity across the studies was quantified using the *I*^2^ statistic, which indicates significant heterogeneity when *I*^2^ > 50% [48]. We also conducted *post hoc* subgroup analyses according to the median number of ovarian cases (≥ 150 *vs*. < 150), country (Asia *vs*. Europe), cut-off method (ROC *vs*. non-ROC), and adjustments made for potential confounders (including age at surgery/diagnosis, FIGO stage, tumor grade, PS, CA-125 level, and other systemic inflammatory response markers). Heterogeneity between subgroups was evaluated by meta-regression analysis. Small study biases (e.g., publication bias) were assessed by visually inspecting a funnel plot analysis and by conducting tests according to Begg et al. [49] and Egger et al. [50]. Sensitivity analyses were conducted by removing one study at a time to examine the effect of data from each study on the overall estimate. All statistical analyses were performed using Stata 12.0 (StataCorp LP).

## SUPPLEMENTARY FIGURES


