# Supplementary material for: Preoperative neutrophil-to-lymphocyte ratio is a predictor of survival of epithelial ovarian cancer: a systematic review and meta-analysis of observational studies
Source: Oncotarget. 2017 Apr 3;8(28):46414–24. doi: 10.18632/oncotarget.16793 (PMC5542277; doi:10.18632/oncotarget.16793)
Supplement: Supplementary file 1 [file oncotarget-08-46414-s001.pdf]

Preoperative neutrophil-to-lymphocyte ratio is a predictor of survival of epithelial ovarian cancer: a systematic review and meta-analysis of observational studies

Supplementary Materials

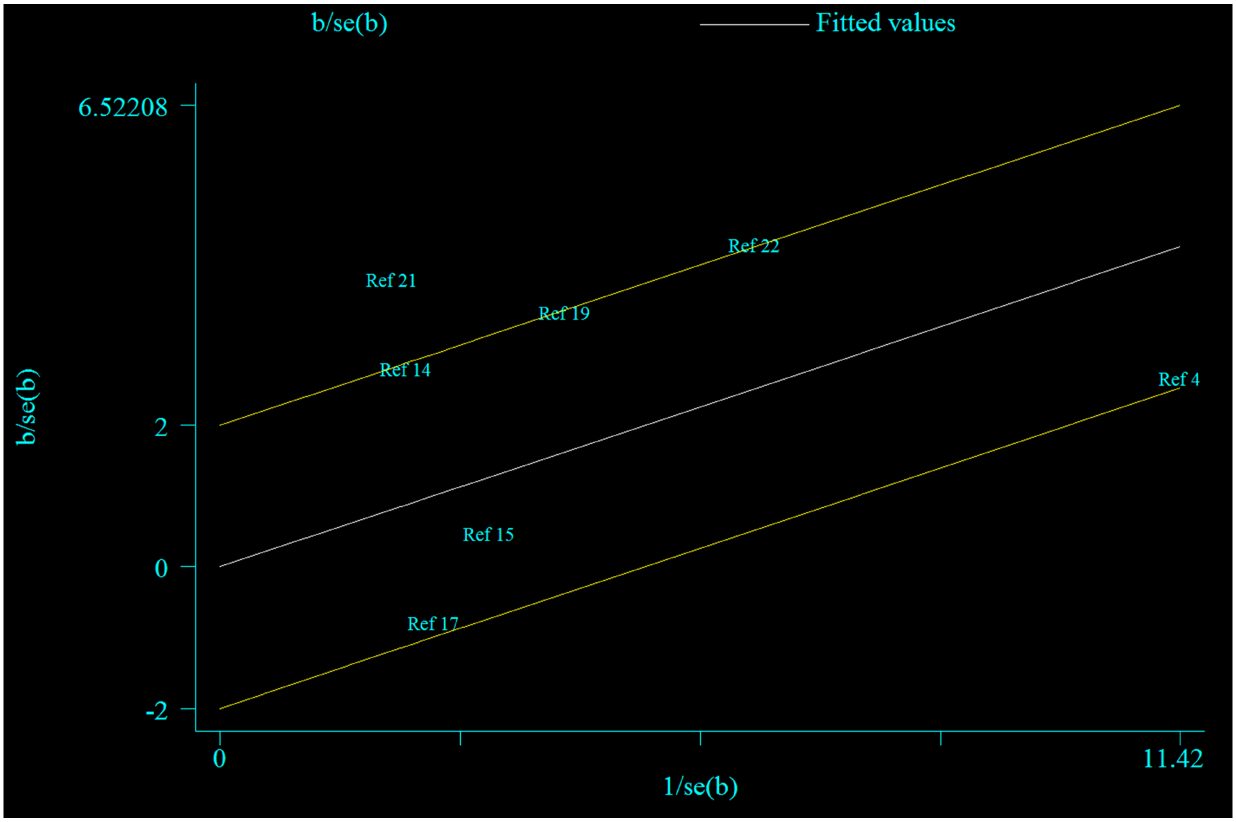

Supplementary Figure 1: Galbraith plot corresponding to the relationship between neutrophil-to-lymphocyte ratio and progression-free survival of patients with ovarian cancer. SE, standard error.

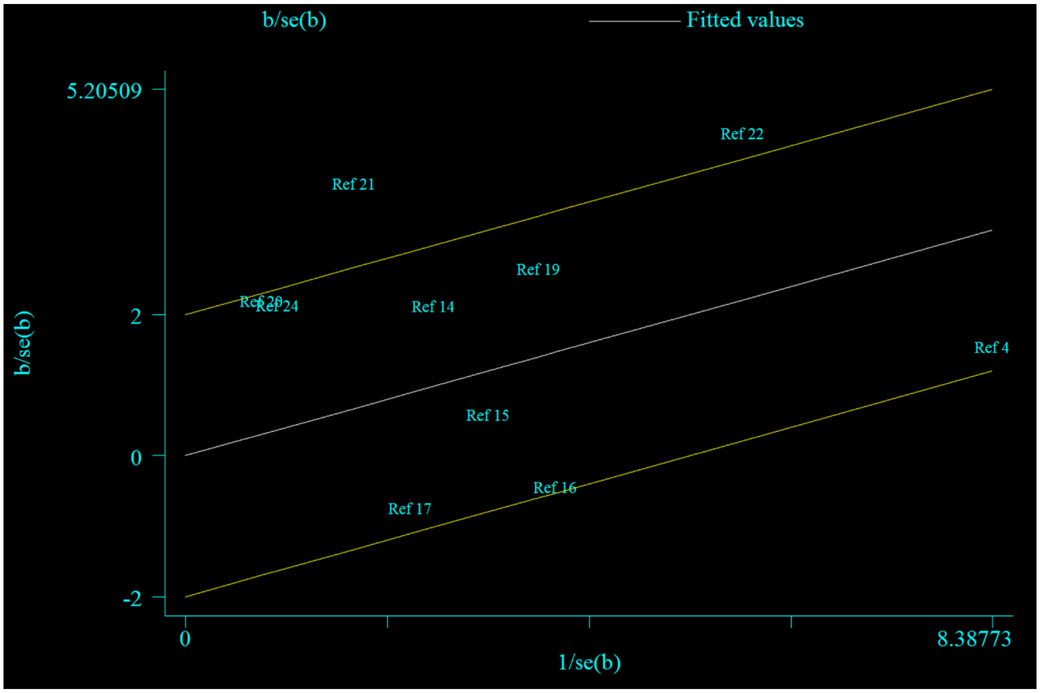

Supplementary Figure 2: Galbraith plot corresponding to the relationship between neutrophil-to-lymphocyte ratio and overall survival of patients with ovarian cancer. SE, standard error.
